# Tongue Lesions in Monkeypox

**DOI:** 10.1590/0037-8682-0386-2024

**Published:** 2025-03-31

**Authors:** Luis Arthur Brasil Gadelha Farias, Karene Ferreira Cavalcante, Ramiro Moreira Tavares

**Affiliations:** 1 Universidade de São Paulo, Departamento de Doenças Infecciosas do Hospital das Clínicas, Laboratório de Investigação Médica - LIM 49, São Paulo, SP, Brasil.; 2 Hospital São José de Doenças Infecciosas, Fortaleza, CE, Brasil.; 3 Centro Universitário Christus, Faculdade de Medicina, Fortaleza, CE, Brasil.; 4 Laboratório Central de Saúde Pública do Ceará, Departamento de Virologia, Fortaleza, CE, Brasil.; 5 Hospital Universitário Walter Cantídeo, Fortaleza, CE, Brasil.

A 29-year-old man was admitted to our emergency department with fever, odynophagia, myalgia, and intense tongue pain. Physical examination revealed five ulcerated lesions on the base of the tongue and one lesion on the dorsal surface, with an umbilical appearance and a whitish crust (Figure 1). The patient presented with bilateral anterior cervical lymphadenopathies and clean aphthous tonsil lesions. The patient had three small erythematous papules on the trunk and soles. Because of a suspected mono-like infection, serologies for Epstein-Barr virus, cytomegalovirus, parvovirus B19, and herpes simplex virus types 1 and 2 were performed, which were nonreactive for IgM. Laboratory examinations revealed leukocytosis with atypical lymphocytes. Immunofluorescence and Western blot tests for human immunodeficiency virus were negative. Real-time polymerase chain reaction of tongue lesion secretion performed using an “in-house” kit was positive for monkeypox. The patient was treated with 100 mg oral tramadol every 4 h for 14 days, which resulted in healing and shedding of the initial umbilicated whitish lesions, evolving into a small cicatricial tongue fissure (Figure 2).

**FIGURE 1:**
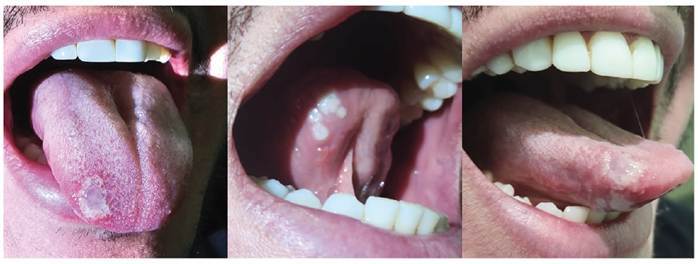
Umbilicated tongue lesions. A large lesion on the dorsum of the tongue with a pox-like appearance, whitish color, and translucent umbilicated center. Five smaller whitish umbilicated lesions are visible near the lingual frenulum on the left side of the tongue. Lateral view shows all tongue lesions.

**FIGURE 2:**
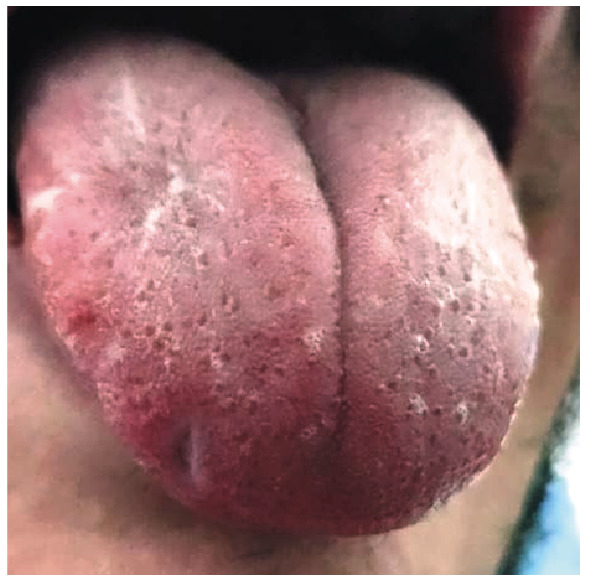
Scarred appearance with a small tongue fissure after 14 days of disease progression and recovery.

Monkeypox is a viral disease of animal origin caused by a Poxviridae virus and closely associated with smallpox viruses^1^ ^,^ ^2^. The oral mucosa may present with lesions that transform from vesicles to pustules, including umbilication and crusting, and may follow the skin around the extremities centrifugally, along with fever and lymphadenopathy, as reported in this case^1^ ^,^ ^3^. Physicians must be aware of the oral manifestations of monkeypox and should examine the oral mucosa to improve patient management and therapy.

## ETHICAL ASPECTS

This case study was approved by the Research Ethics Committee of Hospital Sao José de Doenças Infecciosas (CAAE Nº 83564424.8.0000.5044). An image consent to publish clinical data was obtained from the patient.
